# Successful management of generalized tetanus in a 12-year old girl without anti-tetanus immunoglobulins: a case report

**DOI:** 10.1093/omcr/omac098

**Published:** 2022-09-26

**Authors:** Arlindo Muhelo, Ahivaldino Zita, Amir Seni, Danys Alvarez Arzuaga, Lee Smith, Damiano Pizzol, Yasmin Al Naber

**Affiliations:** Department of Paediatry, Central Hospital of Beira, Beira, Mozambique; Department of Paediatry, Central Hospital of Beira, Beira, Mozambique; Department of Paediatry, Central Hospital of Beira, Beira, Mozambique; Department of Paediatry, Central Hospital of Beira, Beira, Mozambique; Centre for Health, Performance, and Wellbeing, Anglia Ruskin University, Cambridge, UK; Operative Research Unit, Doctors with Africa Cuamm, Beira, Mozambique; Department of Child Health, St John and Paul Hospital, Venice, Italy

## Abstract

In low-income countries tetanus is a major public health concern owing to a low immunization coverage and unclean birth practices. Although it is a vaccine-preventable disease, tetanus can be contracted across the life course. The disease is prevalent and harmful in newborn babies and their mothers when the mothers` are unvaccinated against tetanus. We report on a case of a 12-year-old girl who presented with general malaise, anorexia, dysphagia, trismus and dehydration, which rapidly developed into severe generalized tetanus and was successfully managed in a low-resource setting without the availability of human anti-tetanus immunoglobulins.

## INTRODUCTION

Exposure to spores of the bacterium, *Clostridium tetani*, found in soil, saliva, dust and manure is the cause of the illness tetanus. The bacteria may enter the body through deep cuts, wounds or burns and subsequently affect the nervous system [1]. Although it is a vaccine-preventable disease, tetanus can be contracted across the lifecourse. The disease is prevalent and harmful among newborn babies and their mothers when the mothers are unvaccinated against tetanus (the vaccine being tetanus toxoid) [1]. In low-income countries, tetanus is a major public health concern owing to low immunization coverage and common unclean birth practices. Owing to limited surveillance systems in low- and middle-income countries, it is difficult to estimate the true burden of tetanus. It is estimated that in 2015, 79% of deaths are due to tetanus, ∼44 612 globally occurred in south Asia and sub-Saharan Africa [2]. More accurate data are available on neonatal tetanus and WHO estimates that in 2018, 25 000 newborns died from neonatal tetanus [1].

The clinical presentation can include jaw and neck cramping commonly known as ‘lockjaw’ or ‘trismus’, muscle spasms often in the back, abdomen and extremities, as well as painful muscle spasms that are often triggered by noise, dysphagia, seizures, headache, fever and sweating and changes in blood pressure or tachycardia [3]. However, the onset of a generalized tetanus infection is not always associated with above described symptoms and its presentation with isolated oropharyngeal symptoms should also be considered in differential diagnosis with more common oropharyngeal infection as peritonsillar abscess [3]. Thus, a rapid correct diagnosis is mandatory as those with tetanus may deteriorate and become critical with symptoms including severe muscle spasms, autonomic dysfunction and/or respiratory failure [4]. Patients with suspected tetanus require wound care, tetanus immunoglobulins and antimicrobials. They should also be placed on an intensive care unit for treatment and monitoring. We report a case on a 12-year-old girl who presented with general malaise, anorexia, dysphagia, trismus and dehydration, which rapidly developed into severe generalized tetanus and was successfully managed in a low-resource setting without the availability of human anti-tetanus immunoglobulins.

## CASE REPORT

**Figure 1:**
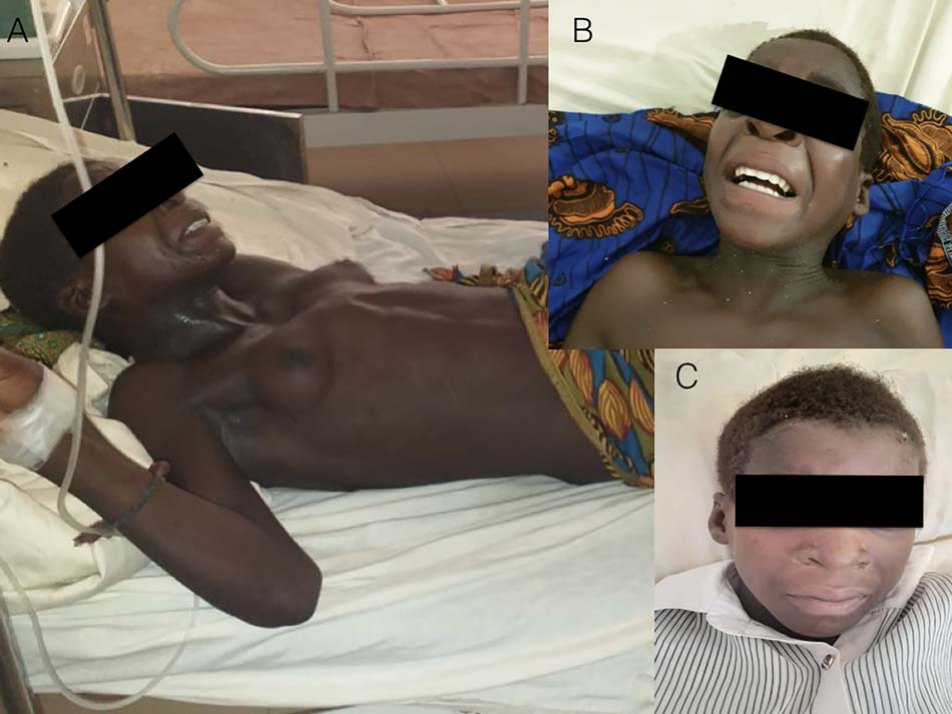
Generalized tetanus in a 12-year-old girl at presentation (**A** and **B**) and at discharge (**C**).

A 12-year-old girl presented with 3 days history of body stiffness at a regional hospital in Mozambique (Fig. 1**A** and **B**). Ten days before she wounded her left thumb with a katana and was treated at a local Health Centre. Three days after the injury, she developed fever and irritability and a week later she developed dysphagia, very painful temporary muscle spasms associated with muscle stiffness, gait changes, asthenia, loss of appetite and increased sweating. The left thumb was oedematous and hyperaemic.

The neurological assessment showed uncontrolled trigeminal, facial and glossopharyngeal nerves with negative Babinski and meningeal signs. The biochemical test performed at admission, after 15 and 30 days are reported in Table 1.

**Table 1:** Biochemical results performed at admission, after 15 and 30 days

Parameter	Admission	15 days after	30 days after
WBC	5.7 × 10^3^/μl	6.3 × 10^3^/μl	5.85 × 10^3^μL
LYM%	40.1%	30.5%	48.0%
NEUT	53.2%	NRA	42.6%
HGB	11.8 g/dl	11.7 g/dl	12.0 g/dL
HTC	39.0%	37.5%	39.4%
MCV	76.3 fL	77.0 fL	78.6 fL
MCH	23.1 pg	24.0 pg	24.0 pg
MCHC	30.3 g/dL	31.2 g/dL	30.5 g/dL
PLT	343 × 10^3^/μL	339 × 10^3^/μL	290 × 10^3^/μL
MBS	Negative	NR	NR
HIV	Negative	NR	NR
Stool parasites	Negative	NR	NR
Urea	5.18 mmol/L	NR	NRA
Creatinine	48.47 μ/L	NR	NRA
Uric acid	154.2 mmol/L	NR	191.0 mmol/L
AST	63.40 IU/L	NR	9.55 IU/L
ALT	51.31 IU/L	NR	8.52 IU/L
Proteins	64.51 g/L	NR	NRA
Albumin	35.0 g/L	NR	35.5 g/L
TB	NRA	NRA	6.81 mmol/L
DB	NRA	NRA	1.30mmo/L
ESR	NRA	NRA	21.00 mm/h

ALT: alanine aminotransferase; AST: aspartate aminotransferase; DB: direct bilirubin; ESR: erythrocyte sedimentation rate; HGB: haemoglobin; HTC: haematocrit; LYM: lymphocytes; MBS: malaria blood smear; MCV: mean corpuscular volume; MCH: mean corpuscular haemoglobin; MCHC: mean cell haemoglobin concentration; NEUT: neutrophils; NR: not required; NRA: not reagents available; PLT: platelets; TB: total bilirubin;WBC: white blood cells

Based on clinical history, examination and test results, the diagnosis of generalized tetanus was made. The girl was isolated and supported with oxygen (0.3 L/min). To control muscle spasms, a combined therapy was started including Diazepam 3 mg/kg per day three times per day and Baclofen 5 mg twice per day. As anti-tetanus immunoglobulins were not available, a combined treatment, including Ceftriaxone 100 mg/kg twice per day, Metronidazole 30 mg/kg three times per day, hydrocortisone 8 mg/kg four times per day and Ranitidine 8 mg/kg twice per day, was administered.

The wound was also cleaned daily and medicated. Finally, two doses of Diphtheria, Tetanus, Pertussis Vaccine were administered, the first dose after 10 days from admission and the second after 30 days from the first dose.

The conditions improved slowly but constantly and she was discharged after 42 days (Fig. 1**C**).

## DISCUSSION

Tetanus still remains a substantial problem, also in adults, in many low- and middle-income countries due to the lack or the disruption of vaccination programmes [5]. As most cases occur in low-income and middle-income countries where surveillance systems are limited, it is difficult to estimate the true burden of tetanus. The appropriate management of generalized tetanus includes prevention of toxin uptake, control of muscle spasms, wound cleaning and debridement and supportive care. The most recognized treatment for the neutralization of tetanospasmin is the human anti-tetanus immunoglobulins [6]. Moreover, the possibility to have a mechanical ventilation support is associated with improved outcomes as it allows spasms to be controlled by high doses of sedatives and, when available, neuromuscular blocking agents [7]. The particularity of this case is the successful management and follow-up of a late stage of generalized tetanus in a low-resource setting with lack or limited tests and treatment availability. Importantly, it was not possible to confirm the diagnosis by any laboratory tests and also routine tests were limited due to the shortage of reagents. Moreover, both the human anti-tetanus immunoglobulins and magnesium sulphate, that are considered a good option in order to reduce the need for other pharmacological agents, were not available [8]. In general, in low-resource settings, the scarcity of resources and appropriately trained staff leads this disease to have a high mortality [9]. In addition to the present case, this is an opportunity to highlight the necessity of rapid and innovative interventions particularly suited to low-resource settings.

It is now mandatory to strengthen and improve health systems both in terms of equipment and public health policies to promote vaccine campaigns and to achieve a vaccine coverage for all preventable diseases aiming to provide adequate health care to all individuals.
